# Dynamic SPECT Assessment of Hepatic Function in Patients With Liver Disease: A Systematic Review and Meta‐Analysis

**DOI:** 10.1111/1754-9485.70166

**Published:** 2026-08-07

**Authors:** Bader Aloufi, Roger Fulton, Kathy Willowson, Peter L. Kench

**Affiliations:** ^1^ Discipline of Medical Imaging Science, Faculty of Medicine and Health The University of Sydney Sydney New South Wales Australia; ^2^ Department of Diagnostic Radiology, College of Applied Medical Sciences Taibah University Madinah Saudi Arabia; ^3^ Department of Medical Physics Westmead Hospital Sydney New South Wales Australia; ^4^ Department of Nuclear Medicine Royal North Shore Hospital Sydney New South Wales Australia; ^5^ Institute of Medical Physics University of Sydney Sydney New South Wales Australia

**Keywords:** dynamic SPECT, dynSPECT/CT, future liver remnant (FLR), liver function, regional hepatic assessment, SPECT

## Abstract

**Introduction:**

Dynamic Single Photon Emission Computed Tomography (dynSPECT) is an advanced functional imaging modality that enables estimation of temporal changes in radiopharmaceutical distribution and physiological function. While dynSPECT has been employed in several clinical applications, it has not been widely applied to liver imaging. This review aims to investigate the role and effectiveness of dynSPECT in assessing hepatic function.

**Methods:**

Following PRISMA guidelines, literature searches were conducted in MEDLINE, PubMed, Scopus, Embase, IEEE, and Web of Science, including all publications available up to December 2025 that assessed liver function using dynSPECT imaging, standard laboratory function tests and clinical scoring systems. Search results were screened by two authors. Included studies were assessed for bias using the QUADAS‐2 quality assessment test tool.

**Results:**

Sixteen eligible studies were incorporated in this review, involving 715 participants, of whom 87% (624) had hepatic diseases. Meta‐analysis revealed correlations between dynSPECT measures of liver function and the laboratory tests, including Hepaplastin (*r* = 0.5471; 95% CI: 0.4523 to 0.6418), Indocyanine Green (ICG) (*r* = −0.461; 95% CI: −0.831 to −0.081), and Bilirubin (*r* = −0.425; 95% CI: −0.714 to −0.14). Furthermore, the comparison between the two dynSPECT parameters and clinical scoring systems, including the Child‐Pugh score, MELD, and the histologic activity index (HAI), has a significant effect on liver disease assessment, with a combined *p*‐value < 0.001.

**Conclusion:**

DynSPECT is a useful tool for the assessment of liver function in patients with hepatic dysfunction, providing results comparable to clinical laboratory function tests and scoring systems.

## 1 Introduction

Liver surgery, transarterial chemoembolisation (TACE), selective internal radiation therapy (SIRT) and targeted radiation therapy are potential liver‐directed therapeutic options for patients with hepatic disease. Selecting the most appropriate hepatic assessment methods is a crucial step for identifying the type of liver disease, choosing a suitable treatment plan, and predicting patient outcomes [1, 2, 3, 4, 5]. The human liver incorporates multiple cell types, including hepatocytes, sinusoidal endothelial cells, and Kupffer cells (KCs), each with unique physiological pathways that perform complex biochemical processes. This multiplicity allows the liver to preserve general health through various processes involving metabolism, protein synthesis, bile generation and detoxification. Indeed, when hepatic cells are injured, the consequences appear clinically as morphological changes, such as in liver cirrhosis, or as physiological changes, such as in liver enzyme levels. Therefore, the term ‘hepatic function assessment’ does not necessarily refer to evaluating the liver's function directly. Instead, it pertains to the clinical outcomes resulting from alterations in underlying cellular functionality [6].

Computed tomography (CT) volumetry, magnetic resonance imaging (MRI) functional and volumetric imaging, laboratory function tests, clinical scoring or grading systems, and nuclear medicine imaging are reported as reliable approaches for liver assessment [7, 8]. CT‐volumetry indirectly estimates regional functionality of the liver by measuring the proposed volume post‐surgery or future liver remnant (FLR). This method assumes homogeneity of liver parenchyma function; however, in patients with compromised liver parenchyma, it may be inhomogeneous, thus requiring other methods for evaluating regional hepatic function [5]. MRI assessment of global and regional liver function and volume is significantly correlated with ^99m^Tc‐mebrofenin hepatobiliary scintigraphy (HBS) in assessing post‐embolisation hypertrophy [9]. Indeed, Gd‐ethoxybenzyl diethylenetriamine pentaacetic acid (Gd‐EOB‐DTPA), an MRI contrast agent, has attracted increasing attention in preoperative liver assessment for its ability to provide both anatomical and functional information about the liver. It has been found that the results of estimating Gd‐EOB‐DTPA uptake using T1 relaxation time significantly correlate with well‐known liver assessment methods, such as blood function tests and clinical scoring systems [10]. However, Gd‐EOB‐DTPA uptake is affected by impaired liver function, such as cirrhosis or biliary obstruction, which decreases signal intensity and reduces the accuracy of liver assessment [11].

Standard laboratory liver function tests can also estimate liver function, either directly or indirectly, by measuring blood levels of liver enzymes such as bilirubin or albumin, or by assessing liver clearance using the indocyanine green (ICG) clearance method [7, 12, 13]. Another method for assessing hepatic function involves using clinical grading systems that incorporate biochemical parameters with clinical symptoms of hepatic dysfunction. For example, Child‐Pugh scoring, the Histologic Activity Index (HAI), the Mayo Risk Score (MRS), and the Model for End‐stage Liver Disease (MELD) score. Although laboratory tests and clinical scoring systems are common tools for assessing liver function, they provide only a global measure of liver function [14, 15, 16, 17, 18, 19].

Scintigraphic procedures assess hepatic function using radiotracers with different mechanisms of biodistribution, e.g., uptake and clearance [7]. For instance, ^99m^Tc galactosyl human serum albumin (^99m^Tc‐GSA) scintigraphy can determine preoperative hepatic volume and evaluate regional liver functional reserve [20]. GSA is taken up by asialoglycoprotein receptors on the hepatocyte cell membrane in the first 30 to 40 min, before being eliminated by endocytosis and lysosomal degradation process [21, 22]. In addition, scintigraphy with ^99m^Tc‐mebrofenin, an iminodiacetic acid (IDA) agent, assesses liver function by measuring radiotracer uptake and clearance [23]. It is mainly extracted from blood by the liver, with rapid clearance and minimal excretion by the kidneys, making it an ideal agent for assessing liver function. Mebrofenin is taken up into hepatocytes by an organic anion‐transporting polypeptide (OATP) and is processed similarly to bilirubin during uptake and elimination. Since albumin is considered the primary transporter protein for this tracer in the blood, the uptake of mebrofenin in the liver is reduced by a low level of albumin [22]. Although both GSA and mebrofenin are used in dynamic hepatic imaging, they have different mechanisms of hepatic uptake and excretion [24].

Dynamic single‐photon emission computed tomography (dynSPECT) is a non‐invasive 3D imaging technique that enables the study of metabolic and other physiological processes within an organ by measuring the temporal variation in radiopharmaceutical concentration. SPECT imaging permits accurate localisation of radiotracer distribution by removing background superimposition associated with planar imaging. Therefore, dynSPECT could be a valuable modality for assessing hepatic function [25, 26]. Furthermore, combining dynSPECT with CT as a hybrid modality (SPECT/CT) enables delineation of the organs of interest by creating an anatomical and attenuation map, which assists with liver volume segmentation and precise radiotracer localisation and quantification. DynSPECT, including dynSPECT/CT, has been employed previously for quantitative functional measurements in brain, heart, renal and lung imaging applications [27, 28, 29, 30, 31, 32]. It is also a reliable method for 3D measurement of hepatic function parameters such as liver uptake ratio and liver uptake density. In addition, it allows the preoperative prediction of post‐operative outcomes after liver surgery [7, 33, 34]. Combining scintigraphic functional assessment with CT volumetric evaluation may provide a more accurate preoperative estimation of liver function. To our knowledge, no systematic review has been conducted to assess the performance of dynSPECT in the functional evaluation of hepatic disease. The aim of this systematic review is to explore the role and effectiveness of dynSPECT in quantifying liver function and its correlation with liver laboratory tests and clinical scoring systems.

## 2 Methods

This review was conducted according to the PRISMA guidelines on literature in the Medline, PubMed, Scopus, Embase, IEEE and Web of Science literature databases (Supporting Information, Table S1a,b) [35]. The search included all published studies up to December 2025 with the following keywords (‘dynamic SPECT‐CT’ OR ‘dynamic SPECT/CT’ OR ‘Dynamic single‐photon emission computed tomography/computed tomography’ OR ‘dynamic SPECT’) AND (haemangiomas OR clearness OR measurement OR assessment OR function*) NOT (cardiac OR myocardi* OR phantom OR animal OR mice), (Supporting Information, Table S2).

### 2.1 Study Selection

The results of the databases' searches were exported to the Covidence Systematic Review Software. Titles and abstracts underwent initial screening by two reviewers, BA and PK [36]. For inclusion, dynSPECT or dynSPECT/CT imaging of the liver that included a SPECT or SPECT/CT acquisition, and to compare dynSPECT imaging results with laboratory function tests and/or clinical scoring results were required. Phantom studies, preclinical experiments, non‐English‐language articles, and studies with insufficient dynSPECT imaging information (such as conference abstracts or short reports) were excluded (Supporting Information, Table S3). The full texts of the remaining manuscripts were then reviewed to select a final set of eligible studies for data extraction. Additionally, the reference lists of the identified studies were searched for any eligible articles not found during the primary search.

### 2.2 Quality Assessment

Separately, BA and PK assessed the quality of the eligible studies using the Quality Assessment Diagnostic Accuracy Studies‐2 (QUADAS‐2) tool, which assesses the ‘risk of bias’ and ‘applicability concerns’ [37].

### 2.3 Data Extraction

The data were extracted into two tables. The first table incorporated the first author's name, publication year, study type, fundamental characteristics of the participants (number, age, sex, and gender), liver diseases in the participants, dynSPECT parameters, comparators (laboratory functional test or clinical score system) and the main findings (the correlation between the dynSPECT parameters and comparator) (Table 1). The second table included the dynamic imaging protocol, such as the type and amount of radiotracer, scanner type, starting time, acquisition time, reconstruction parameters, and use of CT (Table 2).

**TABLE 1:** The characteristics of participants from the eligible studies.

Authors/year	Study type	Participants	Age range (years)	Sex (M/F)	Liver disease	DynSPECT parameters	Comparator		Findings
							Laboratory functional test	Clinical scoring system	
Hino et al. 1997 [38]	P	47	38–74	34/13	HCC (*n* = 38) CCA (*n* = 6) IBDs (*n* = 1) MLC (*n* = 2) Hepatitis *n* = 12 Cirrhosis *n* = 26	*K*‐value	Bilirubin, Albumin Prothrombin time. ICG R15	—	The *K*‐value correlated with ICG R15, Bilirubin, Albumin and Prothrombin time, and the correlation with ICG R15 was high.
Kira, Taniguchi, and Takahashi 1998 [39]	P	32	45–82	23/9	HCC (*n* = 32)	Regional KU Regional LHL15	Bilirubin, Albumin Cholinesterase Platelet count. GOT and GPT	—	The laboratory results for the whole liver after chemolipiodolization increased in contrast to before the treatment. The dynamic Parameters KU and LHL15 illustrated change before and after chemolipiodolisation in the whole liver area and segmental parts.
Hwang et al. 1999 [40]	*p*	114	—	—	HCC (*n* = 54) CLD (*n* = 63) MLC (*n* = 22) CCA (*n* = 13) GBC (*n* = 12) BT (*n* = 6) Hepatitis (*n* = 1)	Total and Regional clearness GSA clearance (*K*‐value)	Bilirubin, Albumin Hepaplastin Cholinesterase ICG15 and K ICG	—	The total GSA clearance Strongly correlated with ICGR15, cholinesterase, serum albumin, and Hepaplastin. The mean GSA clearance was significantly correlated with ICG R15, esterase, serum albumin, and Hepaplastin. GSA clearance and total bilirubin not correlated.
Yamakado et al. 2001 [33]	*p*	27	24–75	16/6	Hepatitis (*n* = 20) BCS (*n* = 2)	Total (Ku) Mean (Ku)	Bilirubin, Albumin Cholinesterase AST and ALT prothrombin time Normo test ICG R15	Child‐Pugh score and HAI	The total Ku value correlated with all Laboratory test except prothrombin time. The mean Ku value correlated with all Laboratory except ALT and albumin. Total and mean Ku were significantly correlated with HAI.
Jonas et al. 2001 [41]	*p*	10	28–43	2/3	PSC (*n* = 5)	C‐max t‐max Excretion (T1/2)	Standard biochemical liver function tests	—	Lower C‐max and long excretion rate t1/2 in patients with severe liver diseases. No correlation between t‐max and the comparative parameters.
Shuke, Aburano, et al. 2003 [42]	P	30	35–71	20/10	CLD (*n* = 23) MLC (*n* = 7)	LU 6 LU15 LU 30	Bilirubin, Albumin Heppapalstin, Prothrombin time Cholinesterase. ICG15	Child‐Pugh score	LU6, LU15 and LU30 were correlated significantly with all Laboratory tests. The significant relation between LU6, LU15 and LU30, and the Child‐Pugh score decreased with the severity and time.
Satoh et al. 2003 [43]	P	57	35–73	34/23	HCC (*n* = 33) CCA (*n* = 9) GBC (*n* = 7) MLC (*n* = 8)	*K*‐value	Bilirubin, Albumin Cholinesterase Prothrombin time Hepaplastin test ICG 15	—	The *K*‐value was slightly correlated with ICG R15, Bilirubin and Hepaplastin test.
Li et al. 2003 [44]	P	54	39–71	37/7	HCC (*n* = 21) MLC (*n* = 16) BT (*n* = 4) CCA (*n* = 3) Hepatitis *n* = 15 Cirrhosis *n* = 6	*K*‐value/FV	—	Hepatitis, Cirrhosis, Disease free parenchyma	The ratio of post to pre‐operative clearance of unit functional volume negatively correlated with the percentage of resection. Increasing the percentage of the reaction will decrease the clearance in unit functional volume.
Shuke, Okizaki, et al. 2003 [45]	P	62	18–27	36/26	HCC (*n* = 27) MLC (*n* = 5) CCA (*n* = 2) Hepatitis *n* = 21 Cirrhosis *n* = 34	Receptor amount Regional uptake	Hepaplastin test, BCAA/TYR, Plasma concentration ratio ICG 15.	Child‐Pugh score	ASGP respecter amount was positively correlated with Hepaplastin test and BCAA/TYR ratio, while was negatively correlated with ICG 15. The dynSPECT parameter with clinical score (child's classification) showed significant difference and decreased with the severity and time.
Jonas et al. 2006 [46]	P	18	22–74	9/9	PSC (*n* = 18)	C‐max t‐max Excretion (T1/2)	Bilirubin, Albumin AST	Child‐Pugh score and Myo risk score	LU6, LU15 and LU30 were correlated significantly with all Laboratory tests. The significant relation between LU6, LU15 and LU30, and the Child‐Pugh score were decreased with the severity and time.
Iida et al. 2009 [47]	R	27	20–68	22/5	HCC (*n* = 16) LF (*n* = 2) Hepatitis (*n* = 18) Cirrhosis (*n* = 7)	LHL15	Bilirubin AST and ALT ALP and GGT Prothrombin time	MELD	The LHL15 negatively correlated with AST, ALT, Bilirubin (T‐Bil) and Prothrombin time 14 days post‐surgery. After one month from the resection the significancy continuous between the LHL15 and AST, PT‐INR, and T‐Bil. There was no correlation between (LHL15) and MELD.
Iimuro et al. 2010 [48]	R	32	46–80	23/9	HCC (*n* = 32)	LHL15 LUR	ICG15 Bilirubin, Albumin Prothrombin time Platelet counts Cholinesterase	HAI	The LHL15 was strongly correlated with LUR of ^99m^Tc GSA. LUR was positively correlated with laboratory test.
Wang et al. 2013 [49]	P	14	44–83	13/1	CCA (*n* = 3) HCC (*n* = 10) MLC (*n* = 1)	HEF	ICG1/2.	—	HEF significantly correlated with ICG in negative direction.
Wang and Cao 2013 [50]	P	14	44–83	13/1	HCC (*n* = 9) CCA (*n* = 3) MLC (*n* = 2)	HEF	ICG	—	HEF significantly correlated with ICG. The mean of HEFs estimated with the vascular input function (VIF) correlated much better with ICG1/2 than using the arterial input function (AIF).
Mao et al. 2015 [34]	R	142	19–72	51/18	HCC (*n* = 47) Haemangioma (*n* = 11) MLC (*n* = 2) CCA (*n* = 3) Other (*n* = 6) LF (*n* = 51) Hepatitis *n* = 51 Cirrhosis *n* = 51	UI	Bilirubin Prothrombin time ICG15	Child scores	The UI negatively correlated with ICG15, prothrombin time and total bilirubin level. UI value was a significant difference between a group of Child scores (5, 6 and group B (from 7 to 8)).
Annamária, Libor et al. 2024 [51]	P	35	35–78	21/14	HCC (*n* = 8) Haemangioma (*n* = 1) MLC (*n* = 18) CCAs (*n* = 6) GBC (*n* = 2) Cirrhosis (*n* = 4)	TLF	ICG15	MELD Child‐Pugh score APRI score	The TLF negatively correlated with ICG15, and MELD, but not with Child‐Pugh score APRI score.

Abbreviations: ALT, alanine transaminase; APRI, aspartate aminotransferase/platelet ratio index; ASGP, asialoglycoprotein receptors; AST, aspartate transaminase; BCAA/TYR, amino acids/tyrosine; BCS, Budd‐Chiari syndrome; BDs, bile duct stone; BT, benign tumour; CCA, Cholangiocellular carcinoma; CLD, chronic liver disease; C‐max, measuring peak of time curve activity; F, female; FV, function liver volume; GBC, gall bladder cancer; GGT, gamma‐glutamyl transferase; GOT and GPT, function liver tests (serum glutamic oxaloacetic transaminase and serum glutamic pyruvic transaminase, respectively); HAI, histologic activity index score; HCC, hepatocellular carcinoma; HEF, volumetric hepatic extraction fraction; ICG R15, indocyanine green test after 15 min; K ICG, plasma clearness rate of ICG; *K*‐value, the clearance of radiotracer from the hepatocytes; LF, liver failure; LHL15, ratio of liver to heart‐plus‐liver radioactivity at 15 min; LU 6, LU15, LU 30 liver uptake at 6, 15 and 30 min after injection of radiotracer; LUR, liver uptake ratio; M, male; MELD, model for end stage liver disease; MLC, metastatic liver cancer; P, prospective study; PSC, primary sclerosing cholangitis; R, retrospective study; Regional KU; regional uptake constant index; TLF, total liver filtration; t‐max, time point at peak of time curve activity; UI, uptake index; V, liver volume.

**TABLE 2:** DynSPECT imaging factors extracted from eligible studies.

The author/years	Radiotracer	Amount of radiotracer (MBq)	SPECT scanner type	Time of starting imaging (min)	Acquisition parameters (time per rotation (s)/number of rotations/total time (min))	Image reconstruction parameters	Filter types in reconstruction	Attenuation correction (yes/No)	Use of CT with SPECT
Hino et al. 1997 [38]	^99m^Tc‐Sn colloid	185	A single head (CCA‐90B (Toshiba Corporation, Tokyo))	0	37/35/21	Matrix size (64 × 64) pixel size (5.4 mm) Slice thickness (1.08 cm) smoothing in pre and post reconstruction (9 points)	—/Shepp and Logan filters	No	No
Kira, Taniguchi, and Takahashi 1998 [39]	^99m^Tc‐GSA	185	A dual head (GCA‐90B (Toshiba Corporation, Tokyo)) with LEHR collimator	0	60/16/16	Matrix size (6 × 64) Slice thickness (3.2 cm)	FBP/Shepp and Logan filters	**—**	No
Hwang et al. 1999 [40]	^99m^Tc‐GSA	185	Three‐head gamma camera system (Toshiba GCA9300A, Toshiba, Nasu, Japan) with LEHR collimator	1	60/15/15	Matrix size (64 × 64) Pixel size (6.4 mm) Slice thickness (1.28 cm)	—/Ramp with Butterworth filters	No	No
Yamakado et al. 2001 [33]	^99m^Tc‐GSA	148	Dual‐head system (GCA‐7200A, Toshiba, Nasu, Japan)		30/40/20	**—**	FBP/Shepp & Logan filters with Butterworth	Yes	Yes
Jonas et al. 2001 [41]	^99m^Tc‐HIDA (etifenin)	120	Triple head gamma camera (Triad XLT, Trionix Inc., Twinsburg, OH, USA) with LEUHR collimator	0	360 per tomo/12/6 m	Matrix size (12 × 128) Slice thickness (0.03 cm)	FBP/Ramp filter with 2‐D Hamming filter	No	No
Shuke, Aburano, et al. 2003 [42]	^99m^Tc‐GSA	185	Triple‐headed, rotating, gamma camera system (GCA9300A and GMS550, Toshiba Medical, Tokyo, Japan)	1	60/30/30	Matrix size (6 × 64) Pixel size (41 mm) Slice thickness (0.64 cm)	FBP/Ramp filter with Butterworth filter	No	No
Satoh et al. 2003 [43]	^99m^Tc‐GSA	185 (3 mg)	Dual head (RC 26001, Hitachi, Tokyo, Japan) with LEHR collimator	0	35/35/20	Matrix size (6 × 64) Pixel size (5.4 mm) Slice thickness (1.08 cm)	Shepp and Logan filters	—	—
Li et al. 2003 [44]	^99m^Tc‐GSA	185	A triple‐head gamma camera (Toshiba GCA9300A, Toshiba, Nasu, Japan) with LEHR collimator	1	60/15/15	Matrix size (6 × 64) Pixel size (6.4 mm) Slice thickness (1.28 cm)	—/Ramp filter with Butterworth filter	No	NO
Shuke, Okizaki, et al. 2003 [45]	^99m^Tc‐GSA	185	Triple‐head rotating gamma camera system (GCA9300A and GMS550; Toshiba Medical, Tokyo, Japan) with LEGP collimator	1	60/30/30	Matrix size (6 × 64) Pixel size (6.4 × 6.4) Slice thickness (0.64 cm)	FBP/Ramp filter with Butterworth filter	No	No
Jonas et al. 2006 [46]	^99m^Tc‐HIDA (etifenin)	120	Triple head gamma camera (Triad XLT, Trionix, Twinsburg, OH, USA) with LEUHR collimator	0	360 per tomo/12/6	Matrix size (12 × 128) slice thickness (3.51 mm^3^)	FBP/Ramp filter with 2‐D Hamming filter	No	No
Iida et al. 2009 [47]	^99m^Tc‐GSA	185	Triple‐headed, rotating, gamma camera system (GCA 7200A; Toshiba, Tokyo, Japan) with LEGP collimator	1	60/30/30	Matrix size (6 × 64) Pixel size (40 mm) Slice thickness (0.64 cm)	FBP/Ramp filter and Butterworth filter	No	No
Iimuro et al. 2010 [48]	^99m^Tc‐GSA	185	A SPECT/PET system (Forte, Philips/ADAC Laboratories, Milpitas, CA) with helical x‐ray CT scanner (Prima, Hitachi Medical Co., Tokyo, Japan) (dual camera) with LEHR collimator	0	60/30/30	Pixel size (5.9 mm)	—	Yes	Yes
Wang et al. 2013 [49]	^99m^Tc‐mebrofenin	370	Hybrid SPECT/CT scanner (Siemens Medical Solutions)	0	—/—/60	Matrix size (12 × 128) Pixel size (500 mm)	FBP/—	Yes	Yes
Wang and Cao 2013 [50]	^99m^Tc‐mebrofenin	370	A Siemens hybrid SPECT/CT scanner (Symbian T6, Simens Medical Solutions) with LEHR collimator	0	20 per stop/64 projections/60	Matrix size (12 × 128)	FBP/Butterworth filter	Yes	Yes
Mao et al. 2015 [34]	^99m^Tc‐GSA	—	SPECT/CT system	—	—	—	—	—	Yes
Annamária, Libor et al. 2024 [51]	^99m^Tc‐mebrofenin	300	Three‐headed integrated (SPECT/CT) (Mediso AnyscanTRIO, Mediso Medical Imaging Systems Ltd., Budapest, Hungary)	0	192‐s/frequency rotation	Matrix size (128 × 128) Pixel size (4.22 mm)	—	Yes	Yes

Abbreviations: FBP, filtered back projection; LEGP, low energy general purpose collimator; LEHR, low energy high‐resolution collimator; LEUHR, low energy ultra high‐resolution collimator.

### 2.4 Statistical Analysis

Microsoft Excel and Jamovi (The Jamovi Project, Version 2.3) software were used for statistical analysis [52]. A meta‐analysis was conducted to assess the agreement between liver dynSPECT and laboratory tests of hepatic function. Studies were divided into subgroups to compare the mean correlations of dynSPECT liver function parameters (Table 1) with Bilirubin, Albumin, ICG, Hepaplastin, Prothrombin time, Cholinesterase, Alanine aminotransferase (ALT) and Aspartate aminotransferase (AST). Continuous variables were reported as means, and categorical data as percentages. A Forest plot with 95% confidence intervals (95% CI) and a Hedges model pooled the correlation coefficients (*r*) for dynSPECT parameters and summarised the effect sizes of studies that compared results with laboratory tests [53]. The interpretation of pooled correlation coefficients was considered very strong if *r* ≥ 0.90, strong if *r* (0.70 to 0.89), moderate if *r* (0.50 to 0.69), slight if r (0.30 to 0.49) and negligible if *r* ≤ 0.29 [54]. Moreover, Fisher's r‐to‐z transformation was used to normalise the variation in correlation coefficients across studies [55]. Heterogeneity (tau^2^) was estimated for all studies using Hedges' estimator [56]. Additionally, the Cochran's test (*Q*‐test), which is a statistical method for estimating heterogeneity, and the percentage of heterogeneity (*I* ^2^ index) were calculated. A heterogeneity percentage (*I* ^2^) above 75% is considered high heterogeneity [57, 58].

Additionally, the Funnel Plot symmetry, Begg and Mazumdar rank correlation, and Egger linear regression tests were used to assess publication bias [59, 60, 61]. Moreover, outliers in the dynamic parameters were identified using studentized residuals. A *p*‐value < 0.05 was considered significant for all tests [62]. Fisher's method was chosen for comparisons of liver dynSPECT parameters with the clinical scoring systems. Most included studies only reported *p*‐values without providing details of the original data. Fisher's combined probability test is a common method for this type of assessment, combining individual *p*‐values from independent investigations and presenting the result as (pcombine‐value) and a Chi‐Square (*χ* ^2^) with degrees of freedom (df) [63].

## 3 Results

### 3.1 Studies and Patient Characteristics

**FIGURE 1:**
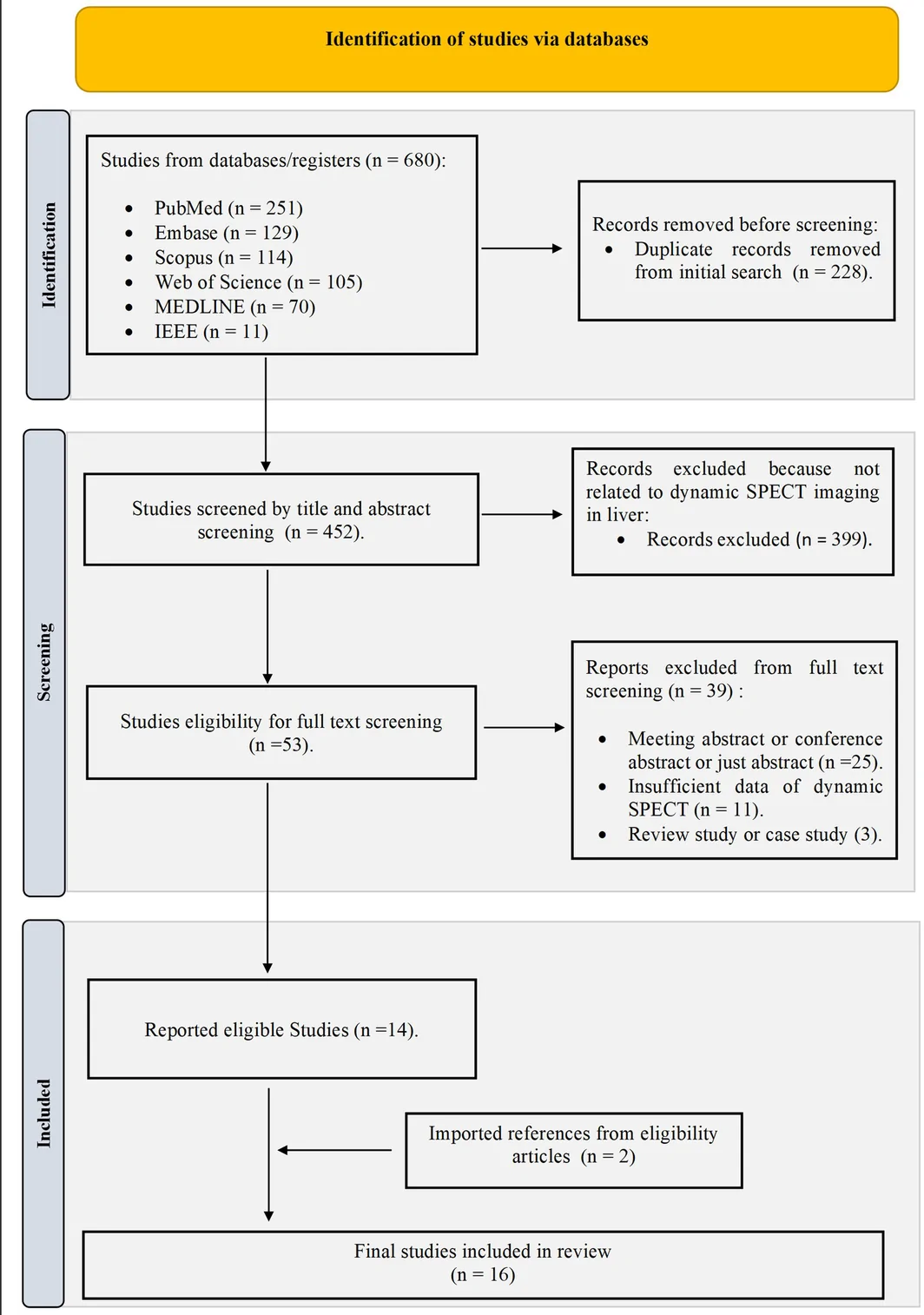
Flowchart of searching method to select eligible studies for dynSPECT imaging.

The initial search yielded 680 potential studies; after removing duplicates, 452 studies moved on to the title and abstract screening stage. Of these, 53 liver imaging studies were eligible for full‐text screening. Two additional studies were added from the reference lists of the eligible results. The full‐text screening identified 16 articles that met the inclusion and exclusion criteria (Figure 1) [33, 34, 38, 39, 40, 41, 42, 43, 44, 45, 46, 47, 48, 49, 50, 51]. These comprised 13 prospective (81%) and 3 retrospective (19%) studies published between 1997 and 2025. They involved 715 participants aged 18 to 83 years, including 354 males and 154 females, as shown in Table 1.

Most participants, 87% (*n* = 624), had liver diseases listed in Table 1. Hepatocellular carcinoma (HCC) was the most common liver disease, affecting 52% (*n* = 327) of participants in twelve of sixteen studies. Ten studies involved participants with metastatic liver cancer (MLC), present in 13% (*n* = 83), while nine studies included patients with cholangiocarcinoma (CCA), making up 8% (*n* = 48). Gallbladder cancer (GBC) was reported in 3% (*n* = 21) across three studies. Liver failure (LF), primary sclerosing cholangitis (PSC), haemangioma, and benign tumour (BT) were identified in two studies, with 9% (*n* = 53), 4% (*n* = 23), 2% (*n* = 12), and 2% (*n* = 10), respectively. Additionally, chronic liver disease (CLD), Budd‐Chiari syndrome (BCS), and bile duct stones (BDs) were reported in individual studies. Patients with hepatitis and cirrhosis appeared in seven and six studies, with percentages of 23% (*n* = 138) and 21% (*n* = 128), respectively. Furthermore, the dynSPECT liver function parameters and the reference comparators reported in the included studies are listed in Table 1. The dynSPECT parameters were compared with laboratory function tests and clinical scoring systems in eight studies (50%). In contrast, in only seven studies (44%), dynSPECT parameters were compared with laboratory function tests alone, and in only one study (6%), with clinical scoring systems alone.

### 3.2 DynSPECT Imaging Factors

The eligible studies employed different equipment and acquisition parameters for dynSPECT imaging (Table 2). SPECT camera configurations consisted of single‐detector systems (*n* = 1, 6%), dual‐detector systems (*n* = 7, 44%), and triple‐detector systems (*n* = 8, 50%). Thirteen studies reported acquisition matrix sizes of 64 × 64 (*n* = 8, 50%) or 128 × 128 (*n* = 5, 31%), while three studies did not specify the matrix size. SPECT/CT was utilised in six of the studies.

The eligible studies utilised four different radiopharmaceuticals, listed in Table 2. The most common was ^99m^Tc‐GSA (*n* = 10), with an average activity of 180 MBq. ^99m^Tc‐HIDA (*n* = 2) and ^99m^Tc‐mebrofenin (*n* = 3) were utilised in five studies, with mean injected activities of 120 and 347 MBq, respectively. Additionally, ^99m^Tc‐Sn colloid (*n* = 1) was used in one study with an injected activity of 185 MBq. Imaging was performed immediately after radiotracer administration or 1‐min post‐injection (*n* = 5) in most studies. Moreover, the total duration for dynamic imaging ranged from 6 to 60 min (Table 2).

Filtered back projection (FBP) was the most common reconstruction method in nine studies, with various filters used, such as Ramp with Butterworth (*n* = 5, 33%), Shepp and Logan (*n* = 3, 20%), Ramp with 2‐D Hamming (*n* = 2, 13%), Shepp & Logan with Butterworth (7%), and Butterworth alone (7%), whereas the remaining studies did not specify the reconstruction method used. Moreover, attenuation correction was performed in 6 of 16 studies (38%).

### 3.3 Quality Assessment

**FIGURE 2:**
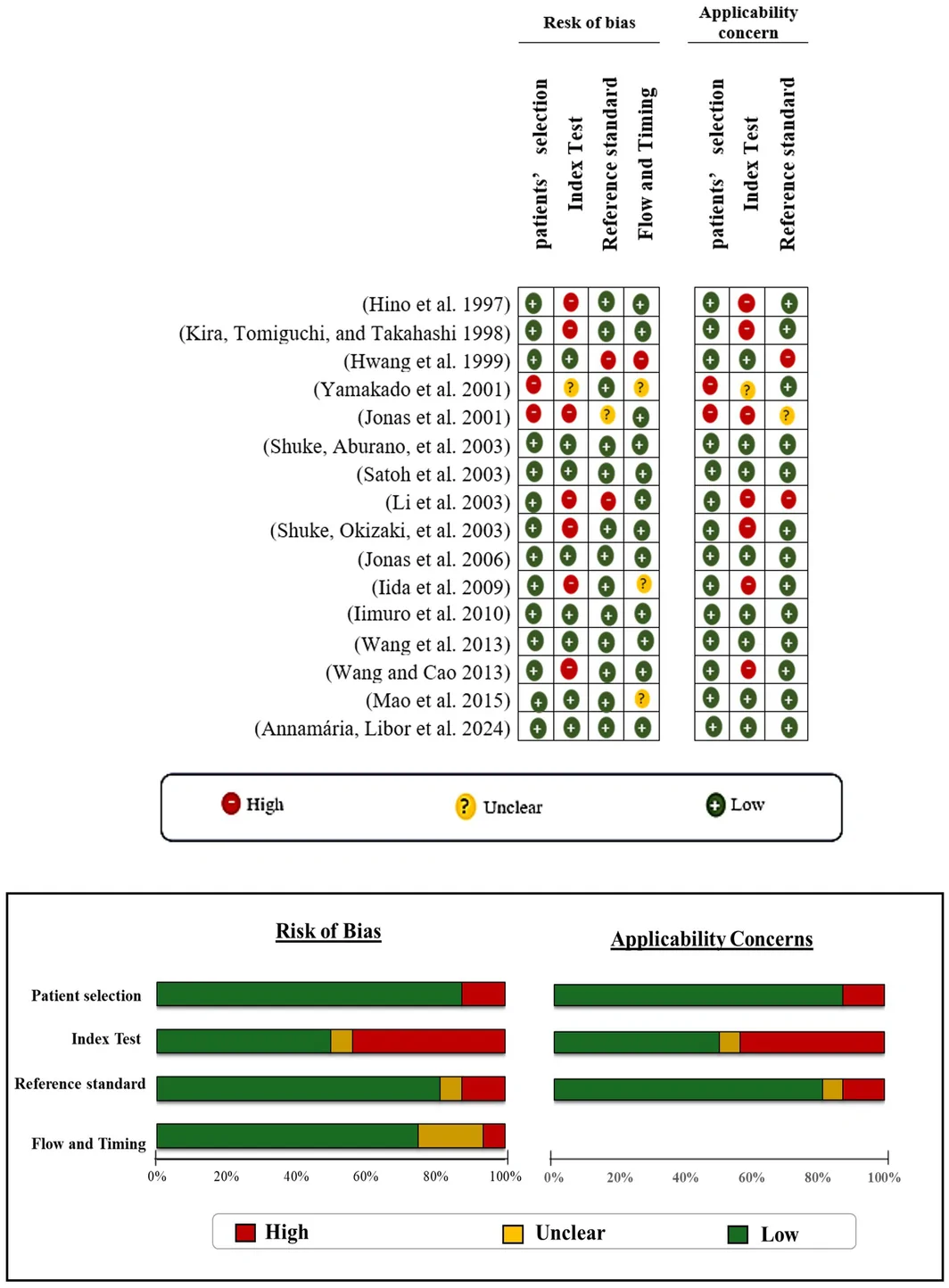
QUADAS‐2 analysis of the risk of bias and the applicability concern.

The results of using the QUADAS‐2 tool to assess risk of bias and applicability concerns are summarised in Figure 2. The index test question yielded results in the mid‐range (~47%) between low and high in terms of risk of bias and applicability concerns. Patient selection and reference standard showed minimal risk of bias and low applicability concerns, with percentages of 88% and 81%, respectively. Similarly, the flow and timing domains had low risk of bias, with about 75% of studies meeting the criteria.

### 3.4 DynSPECT Functional Parameters Compared With Clinical Scoring Systems

During data extraction, clinical scoring systems were reported in eight of the 16 eligible studies, as in Table 1. The functions and roles of these clinical scoring systems in liver assessment are detailed in Table 3. The Fisher's combined probability test was used to assess associations between dynSPECT measures of liver function and clinical scoring system measures, including Child‐Pugh (classes A, B, and C) and histologic activity index (HAI) scores. However, the Fisher method could not be applied to the ABRI and Mayo risk scoring systems, as each was used in only one study. From the Fisher method, the results between dynSPECT parameters and Child‐Pugh were (*χ* ^2^ (10) = 61.15, *p* < 0.00001), between dynSPECT parameters and HAI were (*χ* ^2^ (6) = 35.1, *p* < 0.000004), and between dynSPECT parameters and MELD were (*χ* ^2^ (6) = 19.52, *p* < 0.0034). These findings indicate evidence of significant effects of both dynSPECT functional parameters and clinical scoring systems on liver disease assessment.

**TABLE 3:** Clinical scoring systems extracted from eligible studies.

Clinical scoring system	The roles in the liver assessment
Child‐Pugh score system	It evaluates the severity of chronic liver diseases by measuring five parameters: bilirubin, albumin, prothrombin time (PT), ascites, and hepatic encephalopathy. Based on these measures, patients are classified into three categories: A (less severe liver dysfunction), B (moderately severe dysfunction), and C (most severe dysfunction) [14].
Histologic Activity Index (HAI)	It is used to evaluate liver inflammation and damage based on liver biopsy results. The results range from 0 to 18, with higher values indicating greater liver damage and hepatic inflammation [18].
Mayo Risk Score (MRS)	It uses several factors, including age, serum bilirubin, and albumin, to assess long‐term survival in patients with chronic hepatic diseases [17].
Model for End‐Stage Liver Disease (MELD)	It assesses the severity of liver disease and estimates short‐term mortality risk by measuring creatinine, bilirubin, and prothrombin time (PT). The MELD score ranges from 6 to 40, with higher values indicating more advanced liver disease and an increased risk of death [19].
Aspartate aminotransferase/platelet ratio index and albumin–bilirubin score (APRI/ALBI)	It is used to assess the severity of liver fibrosis [64].

### 3.5 Meta‐Analysis of Liver DynSPECT Functional Parameters Compared With Laboratory Function Tests

**FIGURE 3:**
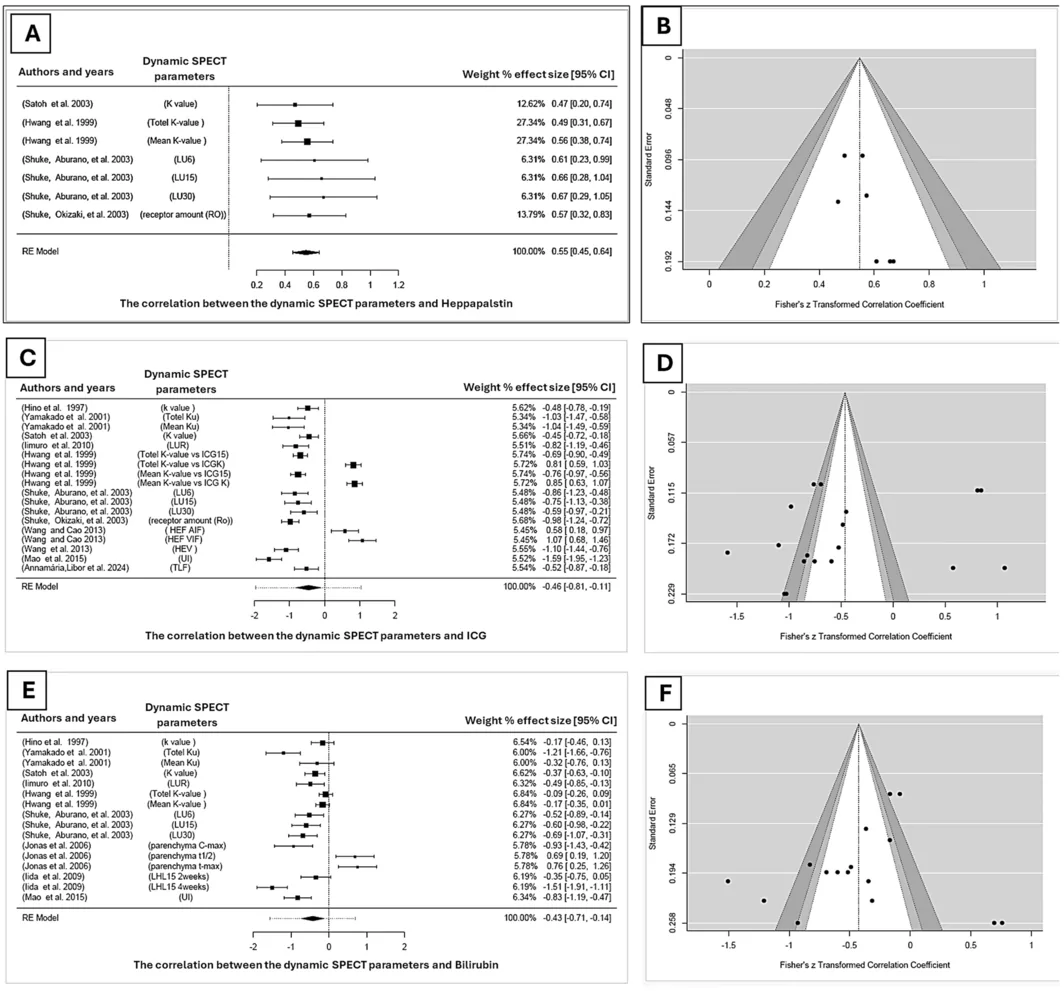
Left column: Forest plots summarising the mean correlation coefficient of dynSPECT parameters with Hepaplastin (A), ICG (C) and Bilirubin (E). Right column: Funnel plots for evaluation of publication bias for Hepaplastin (B), ICG (D) and Bilirubin (F).

A meta‐analysis was conducted to evaluate the effectiveness of dynSPECT‐derived measures of liver function in (Table 1) compared to laboratory function tests. DynSPECT liver functional parameters were significantly correlated with Hepaplastin (*p* < 0.0001), ICG (*p* = 0.010), and Bilirubin (*p* < 0.004), while correlations with other laboratory function tests (Table S4, Supporting Information) were not significant. The pooled correlation coefficient between Hepaplastin and dynSPECT liver function parameters was positive (Figure 3A), and negative for ICG and Bilirubin (Figure 3C,E). Moreover, Cochrane's Q‐test demonstrated low heterogeneity within the Hepaplastin group. In contrast, there was heterogeneity among both the ICG and Bilirubin groups (*I* ^2^ = 96%) and (*I* ^2^ = 92%), respectively. Additionally, there was no evidence of outliers in the Hepaplastin, ICG, and Bilirubin groups, as all studies in each group fell within the threshold range when measured by the Studentized Residuals method (±2.6901, ±2.9738, and ±2.99, respectively). Furthermore, publication bias in the Hepaplastin, ICG, and Bilirubin groups was evaluated using rank correlation and regression tests. Asymmetry in the Hepaplastin funnel plot (Figure 3B) indicated the presence of publication bias with rank correlation (*p* = 0.0390), while no bias was detected with the regression test (*p* = 0.3967). There was no evidence of publication bias in the ICG and Bilirubin funnel plots (Figure 3D,F).

## 4 Discussion

To our knowledge, this systematic review is the first to examine the effectiveness of dynSPECT imaging for hepatic function assessment compared with laboratory tests and clinical scoring systems. Over the past two decades, various methods for assessing hepatic function have been explored, including CT volumetry, MRI combined with functional and volumetric assessment, hepatobiliary scintigraphy (HBS), liver function blood tests, and clinical scoring systems.

This review focused on dynSPECT, which has demonstrated reliability and clinical effectiveness for quantifying both global and regional liver function in patients with various liver diseases and for several clinical evaluation purposes [7, 8]. In this review, dynSPECT has been used for various purposes in eligible studies. For example, dynSPECT imaging is used for preoperative hepatic assessment, where it has been found useful for estimating liver function after surgery and assessing potential post‐resection risks. Moreover, dynSPECT has been quantitatively useful for estimating regional hepatic reserve post‐chemoembolization. DynSPECT has also played a key role in assessing graft function and predicting post‐surgical outcomes in transplant recipients, as well as evaluating regeneration in liver donors. Additionally, dynSPECT has demonstrated the ability to predict liver function after radiation therapy (RT) and thus assess the potential risk of hepatic damage [33, 34, 38, 39, 40, 41, 42, 43, 44, 45, 46, 47, 48, 49, 50].

This review shows that various dynamic parameters obtained from dynSPECT imaging can contribute to hepatic function assessment (Table 1). Each parameter can indicate multiple aspects of hepatic evaluation; for example, the *K*‐value, which represents the rate of radiotracer clearance from hepatocytes, was significantly correlated with ICG and several laboratory function tests. Moreover, the *K*‐value combined with the functional liver volume (FV) from SPECT or the liver volume (V) from CT, as demonstrated by Hino, Tamai et al. 1997, Satoh, Yamamoto et al. 2003, enabled the estimation of the predictive index value for post‐hepatic surgery, which is considered to be above 0.35 for safe hepatic resection. This indicated that functional volumetric data derived from dynSPECT could extend the assessment possibilities, helping to estimate risk and predict postoperative complications such as PHLF [38, 43]. Another parameter highlighted by this review's findings is liver filtration function across both the global and regional areas. It facilitates pre‐surgical assessment, and as demonstrated by Annamaria et al., dynSPECT can measure liver filtration function in both the overall and regional liver regions, supporting pre‐surgical evaluation. Their study showed a strong correlation between total liver function (TLF) and ICG‐R15 [51].

As shown in Table 1, the dynSPECT binding rate constant (KU) for both global and regional uptake is an additional dynamic parameter identified in this review, reflecting the elimination rate of radiotracers from the liver and the abundance of asialoglycoprotein (ASGP) receptors, which are vital to hepatocyte metabolism. KU demonstrated significant findings when evaluating regional liver dysfunction and may predict future remnant liver function before surgery. Additionally, in this review, LHL15 is a dynamic parameter indicating radiotracer accumulation in hepatic lobes, estimating overall and regional hepatic function to assess graft regeneration and the liver's functional reserve. In fact, LHL15 has been shown to be significantly correlated with KU and standard laboratory tests [33, 39, 47].

Liver uptake ratio (LUR) and volumetric hepatic extraction fraction (HEF) are additional valuable dynamic measures for assessing hepatic function, as reported in this review from eligible studies (Table 1). Indeed, LUR was found to provide information on hepatic functional reserve, indicating the number of hepatocytes present in the whole liver or in a regional area. In contrast, the volumetric hepatic extraction fraction (HEF) was used to assess liver function by analysing the voxel‐by‐voxel spatial distribution of radiotracers. Both LUR and HEF were significantly correlated with ICG. Furthermore, LUR was associated with the Child‐Pugh score, and this correlation appeared to decrease with increasing disease severity and over time [42, 48, 49, 50] Furthermore, Table 1 demonstrates that several quantitative measurements for the time‐activity curve (TAC) can be generated via dynSPECT imaging, including peak counts, time to peak, and excretion half‐time (T1/2). These are useful for monitoring blood flow throughout the liver and its regions. Therefore, detecting hepatic diseases and providing information about liver function [41, 46].

The results of dynamic parameters in this review were compared with well‐established evaluation methods, such as laboratory blood tests and clinical scoring systems. Measuring liver enzymes (bilirubin, albumin, ICG, Hepaplastin, prothrombin time, cholinesterase, ALT, and AST) helps assess liver function. Variations in these enzymes' normal levels may indicate hepatic dysfunction [7, 12]. For example, bilirubin metabolism occurs within hepatocytes; therefore, an increase in blood enzyme levels indicates hepatic cell injury and indirectly suggests liver dysfunction [8]. Furthermore, a decrease in blood levels of albumin, cholinesterase, and hepaplastin, which are synthesised by liver cells, could also signal liver dysfunction. Similarly, ALT and AST are key indicators of liver damage because, under normal conditions, these enzymes are abundant in hepatocytes, but their blood levels are low. When liver function is impaired, the bloodstream levels of ALT and AST rise above normal [14, 65]. Furthermore, dynamic measurement of ICG clearance is commonly used to predict post‐hepatectomy liver failure (PHLF) and postoperative complications, as well as to confirm cirrhosis and HCC in patients [13]. However, it provides only a general assessment of liver function and is less precise in non‐cirrhotic patients [7].

Based on the meta‐analysis results in this review, dynSPECT‐driven measures show slight correlations with Bilirubin (*r* = −0.425) and ICG (*r* = −0.461), and a moderate correlation with Hepaplastin (*r* = 0.5471). This suggests that dynSPECT could provide insights into liver function by measuring dynamic radiopharmaceutical uptake or clearance. Although correlations are moderate to slight across these methods, this emphasises the value of using dynSPECT for liver function evaluation.

Most studies included in this review compared individual liver function tests with dynSPECT‐derived measures. However, in recent years, new blood test systems have been implemented to evaluate liver function and predict post‐surgery based on combinations of blood tests, such as aspartate aminotransferase/platelet ratio index and albumin–bilirubin grade (APRI/ALBI). This systematic review included one study by Annamária, Libor et al. (2024), which found no significant correlation between the dynSPECT measure (TLF) and the APRI score [51]. Thus, the comparability of our findings may be impacted by these new systems, and we suggest that future studies consider adopting these standardised indices to potentially enhance both clinical relevance and methodological consistency [64].

Eight included studies discussed clinical scoring systems that demonstrated good performance in evaluating patients with various liver diseases. For instance, Child‐Pugh aids in selecting patients with HCC and liver cirrhosis for hepatectomy or transplantation. MELD and Mayo Risk predict short‐term survival for liver donor patients, while the HAI provides histological information about the liver parenchyma [15, 16, 17, 66]. This review found evidence of a correlation between dynSPECT parameters and clinical scoring systems (Child‐Pugh, MELD, and HAI) for liver disease assessment, with the Fisher method indicating that the combined *p*‐values were less than 0.001. Consequently, dynSPECT imaging may help identify the type of liver disease and evaluate the condition of the liver parenchyma.

Various dynSPECT imaging protocols were utilised in the studies included in this review (Table 2). Mao, Du et al. (2015) reported that fusing dynSPECT with CT significantly enhanced the anatomical and regional assessment of hepatic function, allowing prediction of future segmental remnant liver function prior to liver resection surgery [34]. This review shows that dynSPECT can be used to assess liver function with various radiotracers, including ^99m^Tc‐GSA, ^99m^Tc‐HIDA, ^99m^Tc‐mebrofenin, and ^99m^Tc‐Sn colloid. Indeed, the liver comprises parenchymal and non‐parenchymal cell types, which have distinct functional processes and mechanisms of uptake and excretion [67, 68]. Therefore, understanding the pathways of these radiotracers is essential for accurate evaluation of liver function and volume. De Graaf, Bennink, et al. (2010) noted that non‐invasive imaging (dynamic planar (dynPlanar) Scintigraphy and static SPECT) plays an important role in assessing hepatic function in surgical and transplantation applications. The authors showed how dynPlanar and static SPECT provide several parameters that benefit the evaluation of both whole and residual liver function [68]. Similarly, dynSPECT can provide dynamic parameters from 3D imaging, quantifying regional function and overcoming limitations of 2D dynPlanar imaging, thereby enhancing liver assessment [69, 70]. Furthermore, new investigations comparing dynSPECT with dynPlanar plus static SPECT, the current standard care imaging protocol, will be important to confirm the value of this modality in remnant liver functional assessment [68].

### 4.1 Limitations

Although this study demonstrates the potential of clinical dynSPECT imaging in assessing hepatic function, it has several limitations. Firstly, this review assessed risk of bias and applicability concerns using the QUADAS‐2. The evaluation indicated a moderate quality in the Index test domain, approximately (~47%). This suggests that in some studies, it was unclear whether the index test was applied consistently across participants or independently of the reference standard, which could increase the risk of bias. Consequently, this may have impacted the reliability of the findings and should be carefully considered when interpreting the results. Secondly, due to the limited number of studies and gaps between them, further subgroup analysis could not be performed, which may limit the generalisability of the results. This review highlights heterogeneity among included studies, particularly in the causes of liver disease, dynamic imaging parameters, and technical aspects such as modalities and tracers. For example, the included studies used different SPECT camera configurations (as shown in Table 2), resulting in differences in imaging speed and quality. Thus, the study's findings may be impacted by inconsistencies among the included studies. For more reliable results, future research should include studies using standardised methodologies to reduce variation in study quality. Thirdly, most of the included studies were conducted about two decades ago, and there is a lack of recent high‐quality prospective research. This highlights the need for well‐designed future studies to confirm our findings accurately. Additionally, the conclusions of this review may be influenced by future advancements in dynSPECT imaging methods and SPECT technology. For instance, the recent introduction of SPECT systems with cadmium‐zinc‐telluride (CZT) detectors has enabled faster acquisition times and better resolution than earlier SPECT systems designs [29].

## 5 Conclusion

DynSPECT is a promising modality for assessing liver function. It provides several dynamic parameters that are valuable for quantifying global and regional liver function. DynSPECT parameters show good correlation with conventional liver assessment methods such as Hepaplastin, ICG, and bilirubin in clinical laboratory tests, as well as with the Child‐Pugh, MELD, and HAI clinical scoring systems. The hybrid dynSPECT/CT technique also enables morphological evaluation of liver volume and offers corrections for quantitative SPECT. Further research is recommended to explore the capabilities of dynSPECT for hepatic assessment, particularly for evaluating new tomographic imaging techniques and technologies.

## Author Contributions

**Bader Aloufi:** conceptualization, methodology, software, validation, formal analysis, investigation, data curation, writing – original draft, writing – review and editing. **Roger Fulton:** conceptualization, methodology, writing – review and editing. **Kathy Willowson:** conceptualization, methodology, writing – review and editing. **Peter L. Kench:** conceptualization, methodology, software, validation, writing – review and editing, supervision, project administration.

## Funding

This study was part of a PhD project at The University of Sydney and sponsored by a PhD scholarship from the Government of the Kingdom of Saudi Arabia, represented by Taibah University.

## Ethics Statement

The authors have nothing to report.

## Consent

The authors have nothing to report.

## Conflicts of Interest

The authors declare no conflicts of interest.

## Supporting information


**Table S1:** (a) PRISMA main checklist. (b) PRISMA abstract checklist.
**Table S2:** The combination of the keywords in all databases.
**Table S3:** The inclusion and exclusion criteria.
**Table S4:** Results of the meta‐analysis that conducted to compare the dynamic parameters with laboratory function test.
**Figure S1:** This representing Figure 3 in the main article, which demonstrates the relationship between dynamic SPECT parameters and Hepaplastin.
**Figure S2:** This representing Figure 3 in the main article, which demonstrates the relationship between dynamic SPECT parameters and ICG.
**Figure S3:** This representing Figure 3 in the main article, which demonstrates the relationship between dynamic SPECT parameters and Bilirubin.

## Data Availability

All data created or analysed through this study are included in this published article and its Supporting Information. In addition, the raw datasets in the current study are available from the corresponding author on reasonable request (Bader Aloufi: bader.aloufi@sydney.edu.au or baoufi@taibahu.edu.sa).
