# Supplementary material for: Dynamic SPECT Assessment of Hepatic Function in Patients With Liver Disease: A Systematic Review and Meta‐Analysis
Source: J Med Imaging Radiat Oncol. 2026 Aug 7;70(6):510–29. doi: 10.1111/1754-9485.70166 (PMC13580416; doi:10.1111/1754-9485.70166)
Supplement: Supplementary file 1 [file ARA-70-510-s001.docx]

**Table 1S.a:** PRISMA Main Checklist

| **Topic** | **No.** | **Item** | **Location where item is reported** |
| --- | --- | --- | --- |
| **TITLE** |  |  |  |
| **Title** | 1 | Identify the report as a systematic review. | Title |
| **ABSTRACT** |  |  |  |
| **Abstract** | 2 | See the PRISMA 2020 for Abstracts checklist |  |
| **INTRODUCTION** |  |  |  |
| **Rationale** | 3 | Describe the rationale for the review in the context of existing knowledge. | Introduction |
| **Objectives** | 4 | Provide an explicit statement of the objective(s) or question(s) the review addresses. | Introduction |
| **METHODS** |  |  |  |
| **Eligibility criteria** | 5 | Specify the inclusion and exclusion criteria for the review and how studies were grouped for the syntheses. | Method; Study selection |
| **Information sources** | 6 | Specify all databases, registers, websites, organisations, reference lists and other sources searched or consulted to identify studies. Specify the date when each source was last searched or consulted. | Method |
| **Search strategy** | 7 | Present the full search strategies for all databases, registers and websites, including any filters and limits used. | Method: Table (2S) in the supplementary |
| **Selection process** | 8 | Specify the methods used to decide whether a study met the inclusion criteria of the review, including how many reviewers screened each record and each report retrieved, whether they worked independently, and if applicable, details of automation tools used in the process. | Method; Study selection; |
| **Data collection process** | 9 | Specify the methods used to collect data from reports, including how many reviewers collected data from each report, whether they worked independently, any processes for obtaining or confirming data from study investigators, and if applicable, details of automation tools used in the process. | Method; Study selection and data extraction |
| **Data items** | 10a | List and define all outcomes for which data were sought. Specify whether all results that were compatible with each outcome domain in each study were sought (e.g. for all measures, time points, analyses), and if not, the methods used to decide which results to collect. | Method; data extraction; |
|  | 10b | List and define all other variables for which data were sought (e.g. participant and intervention characteristics, funding sources). Describe any assumptions made about any missing or unclear information. | Method; data extraction; |
| **Study risk of bias assessment** | 11 | Specify the methods used to assess risk of bias in the included studies, including details of the tool(s) used, how many reviewers assessed each study and whether they worked independently, and if applicable, details of automation tools used in the process. | Method; Quality assessment; |
| **Effect measures** | 12 | Specify for each outcome the effect measure(s) (e.g. risk ratio, mean difference) used in the synthesis or presentation of results. | Method; Statistical analysis; |
| **Synthesis methods** | 13a | Describe the processes used to decide which studies were eligible for each synthesis (e.g. tabulating the study intervention characteristics and comparing against the planned groups for each synthesis (item 5)). | Method; Statistical analysis; |
|  | 13b | Describe any methods required to prepare the data for presentation or synthesis, such as handling of missing summary statistics, or data conversions. | Null |
|  | 13c | Describe any methods used to tabulate or visually display results of individual studies and syntheses. | Null |
|  | 13d | Describe any methods used to synthesize results and provide a rationale for the choice(s). If meta-analysis was performed, describe the model(s), method(s) to identify the presence and extent of statistical heterogeneity, and software package(s) used. | Method; Statistical analysis; |
|  | 13e | Describe any methods used to explore possible causes of heterogeneity among study results (e.g. subgroup analysis, meta-regression). | Method; Statistical analysis; |
|  | 13f | Describe any sensitivity analyses conducted to assess robustness of the synthesized results. | Method; Statistical analysis; |
| **Reporting bias assessment** | 14 | Describe any methods used to assess risk of bias due to missing results in a synthesis (arising from reporting biases). | Method; Statistical analysis; |
| **Certainty assessment** | 15 | Describe any methods used to assess certainty (or confidence) in the body of evidence for an outcome. | Method; Statistical analysis; |
| **RESULTS** |  |  |  |
| **Study selection** | 16a | Describe the results of the search and selection process, from the number of records identified in the search to the number of studies included in the review, ideally using a flow diagram. | Results; Studies and Patient Characteristics; and (Figure 1) |
|  | 16b | Cite studies that might appear to meet the inclusion criteria, but which were excluded, and explain why they were excluded. | Results; Figure 1; |
| **Study characteristics** | 17 | Cite each included study and present its characteristics. | Results; (Table 1). |
| **Risk of bias in studies** | 18 | Present assessments of risk of bias for each included study. | Results; quality assessment and Figure 2; |
| **Results of individual studies** | 19 | For all outcomes, present, for each study: (a) summary statistics for each group (where appropriate) and (b) an effect estimates and its precision (e.g. confidence/credible interval), ideally using structured tables or plots. | Results; (Table 1 and 2) |
| **Results of syntheses** | 20a | For each synthesis, briefly summarise the characteristics and risk of bias among contributing studies. | Results; Quality assessment |
|  | 20b | Present results of all statistical syntheses conducted. If meta-analysis was done, present for each the summary estimate and its precision (e.g. confidence/credible interval) and measures of statistical heterogeneity. If comparing groups, describe the direction of the effect. | Results; Figure (3 ); and (Table 4S) in the supplementary |
|  | 20c | Present results of all investigations of possible causes of heterogeneity among study results. | Results; (Table 4S) in the supplementary |
|  | 20d | Present results of all sensitivity analyses conducted to assess the robustness of the synthesized results. | Results; page; 10 and 11 |
| **Reporting biases** | 21 | Present assessments of risk of bias due to missing results (arising from reporting biases) for each synthesis assessed. | null |
| **Certainty of evidence** | 22 | Present assessments of certainty (or confidence) in the body of evidence for each outcome assessed. | Results; (Table 4S) in the supplementary |
| **DISCUSSION** |  |  |  |
| **Discussion** | 23a | Provide a general interpretation of the results in the context of other evidence. | Discussion; all Discussion |
|  | 23b | Discuss any limitations of the evidence included in the review. | Discussion; Limitations part |
|  | 23c | Discuss any limitations of the review processes used. | Discussion; Limitations part |
|  | 23d | Discuss implications of the results for practice, policy, and future research. | Discussion (conclusion part); |
| **OTHER INFORMATION** |  |  |  |
| **Registration and protocol** | 24a | Provide registration information for the review, including register name and registration number, or state that the review was not registered. | The review was not registered |
|  | 24b | Indicate where the review protocol can be accessed, or state that a protocol was not prepared. | Method: (Table 2S) in the supplementary |
|  | 24c | Describe and explain any amendments to information provided at registration or in the protocol. | null |
| **Support** | 25 | Describe sources of financial or non-financial support for the review, and the role of the funders or sponsors in the review. | null |
| **Competing interests** | 26 | Declare any competing interests of review authors. | In Declarations section |
| **Availability of data, code and other materials** | 27 | Report which of the following are publicly available and where they can be found template data collection forms; data extracted from included studies; data used for all analyses; analytic code; any other materials used in the review. | See Data Availability in Declarations section |

**Table 1S.b:** PRISMA Abstract Checklist

| **Topic** | **No.** | **Item** | **Reported?** |
| --- | --- | --- | --- |
| **TITLE** |  |  |  |
| **Title** | 1 | Identify the report as a systematic review. | Yes |
| **BACKGROUND** |  |  |  |
| **Objectives** | 2 | Provide an explicit statement of the main objective(s) or question(s) the review addresses. | Yes |
| **METHODS** |  |  |  |
| **Eligibility criteria** | 3 | Specify the inclusion and exclusion criteria for the review. | No |
| **Information sources** | 4 | Specify the information sources (e.g. databases, registers) used to identify studies and the date when each was last searched. | Yes |
| **Risk of bias** | 5 | Specify the methods used to assess risk of bias in the included studies. | Yes |
| **Synthesis of results** | 6 | Specify the methods used to present and synthesize results. | No |
| **RESULTS** |  |  |  |
| **Included studies** | 7 | Give the total number of included studies and participants and summarise relevant characteristics of studies. | Yes |
| **Synthesis of results** | 8 | Present results for main outcomes, preferably indicating the number of included studies and participants for each. If meta-analysis was done, report the summary estimate and confidence/credible interval. If comparing groups, indicate the direction of the effect (i.e. which group is favoured). | Yes |
| **DISCUSSION** |  |  |  |
| **Limitations of evidence** | 9 | Provide a brief summary of the limitations of the evidence included in the review (e.g. study risk of bias, inconsistency and imprecision). | No |
| **Interpretation** | 10 | Provide a general interpretation of the results and important implications. | Yes |
| **OTHER** |  |  |  |
| **Funding** | 11 | Specify the primary source of funding for the review. | No |
| **Registration** | 12 | Provide the register name and registration number. | No |

*From:* Page MJ, McKenzie JE, Bossuyt PM, Boutron I, Hoffmann TC, Mulrow CD, et al. The PRISMA 2020 statement: an updated guideline for reporting systematic reviews. MetaArXiv. 2020, September 14. DOI: 10.31222/osf.io/v7gm2. For more information, visit: <www.prisma-statement.org>

| N | Database | Configuration of search strategies |
| --- | --- | --- |
| 1 | PubMed | (“dynamic SPECT-CT " OR ( dynamic adj3 spect ) OR "dynamic SPECT/CT" OR "Dynamic single-photon emission computed tomography/computed tomography" OR "dynamic SPECT" ) AND ( haemangiomas OR clearness OR measurement OR assessment* OR function* ) NOT ( cardiac OR myocardi* OR phantom OR animal OR mice ). |
| 2 | Embase | (“dynamic SPECT-CT " OR ( dynamic adj3 spect ) OR "dynamic SPECT/CT" OR "Dynamic single-photon emission computed tomography/computed tomography" OR "dynamic SPECT" ) AND ( haemangiomas OR clearness OR measurement OR assessment* OR function* ) NOT ( cardiac OR myocardi* OR phantom OR animal OR mice ). |
| 3 | Scopus | (TITLE-ABS-KEY ( "dynamic SPECT-CT " OR ( dynamic W/3 spect ) OR "dynamic SPECT/CT" OR "Dynamic single-photon emission computed tomography/computed tomography" OR "dynamic SPECT" ) AND TITLE-ABS-KEY ( haemangiomas OR clearness OR measurement OR assessment* OR function* ) AND NOT TITLE-ABS-KEY ( cardiac OR myocardi* OR phantom OR animal OR mice ) ) AND ( LIMIT-TO ( LANGUAGE , "English" ) ) |
| 4 | web of science. | (((ALL=(("dynamic SPECT-Ct " OR ( dynamic ads3 spect ) OR "dynamic SPECT/CT" OR "Dynamic single-photon emission computed tomography/computed tomography" OR "dynamic SPECT" ))) AND ALL=(haemangiomas OR clearness OR measurement OR assessment* OR function* )) NOT ALL=(cardiac OR myocardi* OR phantom OR animal OR mice)). |
| 5 | Medline | ( "dynamic SPECT-CT " OR ( dynamic adj3 spect ) OR "dynamic SPECT/CT" OR "Dynamic single-photon emission computed tomography/computed tomography" OR "dynamic SPECT" ) AND ( haemangiomas OR clearness OR measurement OR assessment* OR function* ) NOT ( cardiac OR myocardi* OR phantom OR animal OR mice ). |
| 6 | In IEEE | ("All Metadata": "dynamic SPECT " OR "dynamic SPECT-CT " OR "All Metadata": " dynamic W/3 spect " OR "All Metadata": "dynamic SPECT/CT" OR "All Metadata": "Dynamic single-photon emission computed tomography/computed tomography" OR "All Metadata": "dynamic SPECT") AND ("All Metadata":haemangiomas OR "All Metadata": clearness OR "All Metadata": measurement OR "All Metadata": assessment* OR "All Metadata": function*) NOT ("All Metadata":cardiac OR "All Metadata": myocardi* OR "All Metadata": phantom OR "All Metadata": animal OR "All Metadata": mice) |
| (SPECT) single-photon emission computed tomography and (CT) computed tomography. The (*) is a wildcard character used to expand the search. In all databases it should choose limit to English language. | | |

**Table 2S:** The combination of the keywords in all databases

| Table 3S: The inclusion and exclusion criteria | |
| --- | --- |
| Inclusion criteria | **Exclusion criteria** |
| 1. A study focused on dynamic SPECT or dynamic SPECT/CT imaging in liver and compared that with laboratory function tests.   OR   2. A study focused on dynamic SPECT or dynamic SPECT/CT imaging in liver and compared that clinical grading results | 1. A study focused on dynamic SPECT imaging or dynamic SPECT/CT imaging in the phantom experiments. 2. A study focused on dynamic SPECT imaging or dynamic SPECT/CT imaging in the liver in preclinical experiments (animal experiments). 3. A study focused on dynamic SPECT imaging or dynamic SPECT/CT imaging but written in non-English. 4. A study focused on dynamic SPECT imaging or dynamic SPECT/CT imaging in in application other than liver (such as cardiac, brain, renal and lung) 5. If the study a conference articles or report study with insufficient information about dynamic SPECT imaging |

| Table 4S: Results of the meta-analysis that conducted to compare the dynamic parameters with laboratory function test. | | | | | | | | | | |
| --- | --- | --- | --- | --- | --- | --- | --- | --- | --- | --- |
| Dynamic parameters with laboratory function test | Pooled correlation coefficient | | Fisher r-to-z transformation  (average outcome of correlation coefficients) | | Heterogeneity | | | | Assessment of publication bias | |
|  | r | Effect size [95% CI] | Z | p value | Q-test | p value | tau^2^ | I^2^ (%) | Rank correlation by Begg and Mazumdar | The regression test by Egger’s |
| Bilirubin* | -0.425 | -0.714 to - 0.14 | - 2.883 | p < 0.004 | Q (15) = 112.7318 | p < 0.0001 | 0.3096 | 92% | p = 0.6646 | p = 0.570 |
| Albumin | 0.183 | - 0.081 to 0.447 | 1.358 | p = 0.174 | Q (12) = 69.8574 | p < 0.0001 | 0.1983 | 88.4% | p = 0.1719 | P = 0.119 |
| ICG* | -0.461 | -0.831 to -0.109 | -2.563 | p = 0.010 | Q (17) = 447.617 | p< 0.0001 | 0.554 | 96% | p = 0.879 | p = 0.326 |
| Hepaplastin* | 0.5471 | 0.4523 to 0.6418 | 11.317 | p < 0.0001 | Q (6) = 1.5763 | p = 0.9543 | 0.000 | 000% | p = 0.0390 | P = 0.396 |
| Prothrombin | -0.213 | -0.528 to -0.1026 | - 1.322 | p = 0.018 | Q (10) = 82.5704 | p < 0.0001 | 0.2477 | 88% | p = 0.7513 | p = 0.733 |
| Cholinesterase | 0.1341 | -0.3827 to 0.6508 | 0.508 | p = 0.611 | Q (8) = 139.9528 | p < 0.0001 | 0.5943 | 96% | p = 0.0742 | p = 0.011 |
| Alanine amniotomies (ALT) | 0.0174 | -0.4775 to 0.5123 | 0.068 | p = 0.945 | Q (3) = 15.5458 | p < 0.0004 | 0.2080 | 82% | p = 0.4386 | p = 0.381 |
| Aspartate amniotomies (AST) | -0.0980 | -0.4928 to 0.2967 | -0.486 | p = 0.626 | Q (6) = 29.9891 | p < 0.0001 | 0.2289 | 81% | p = 1.0000 | p = 0.458 |
| * Tests were significantly correlated with dynamic SPECT measures. | | | | | | | | | | |

**Figure 1S:** This representing **Figure 3** in the main article, which demonstrates the relationship between dynamic SPECT parameters and Hepaplastin. The Forest plot in **(A)** provides summary for the mean correlation coefficient of dynamic SPECT parameters with Hepaplastin and shows the effect size [95% confidence intervals] of each correlation coefficient of dynamic SPECT factors. In **(B)**, the Funnel plot that illustrate the effect size of Fisher r-to-z transformation correlation coefficient of dynamic SPECT parameters against standard error to evaluate the chance of publication bias and the black dots in the Funnel plot represent each study.

A

B

A

B

**Figure 2S:** This representing **Figure 3** in the main article, which demonstrates the relationship between dynamic SPECT parameters and ICG. The Forest plot in **(A)** provides summary for the mean correlation coefficient of dynamic SPECT parameters with ICG and shows the effect size [95% confidence intervals] of each correlation coefficient of dynamic SPECT factors. In **(B)**, the Funnel plot that illustrate the effect size of Fisher r-to-z transformation correlation coefficient of dynamic SPECT parameters against standard error to evaluate the chance of publication bias and the black dots in the Funnel plot represent each study.

A

B

**Figure 3S:** This representing **Figure 3** in the main article, which demonstrates the relationship between dynamic SPECT parameters and Bilirubin. The Forest plot in **(A)** provides summary for the mean correlation coefficient of dynamic SPECT parameters with Bilirubin and shows the effect size [95% confidence intervals] of each correlation coefficient of dynamic SPECT factors. In **(B)**, the Funnel plot that illustrate the effect size of Fisher r-to-z transformation correlation coefficient of dynamic SPECT parameters against standard error to evaluate the chance of publication bias and the black dots in the Funnel plot represent each study.
